# Breaking the Silent Barrier: Engineering Antibodies for Brain-Targeted Rabies Immunotherapy

**DOI:** 10.3390/pharmaceutics18070848

**Published:** 2026-07-12

**Authors:** Chen Sun, Liang Xiao, Xiao Guo, Wenxin Wang, Kaihong Zhang, Lei Wang, Zhiqiang Lin, Zhaohui Tang

**Affiliations:** 1 Department of Trauma Surgery, Wuhan Jinyintan Hospital, Tongji Medical College, Huazhong University of Science and Technology, Hubei Provincial Hospital for Infectious Diseases, Wuhan 430023, China; sunchen@hust.edu.cn (C.S.); a364215081@icloud.com (L.X.); shyyyyxy@hotmail.com (X.G.); peato220@outlook.com (W.W.); 15623212575@163.com (K.Z.); byycahs@163.com (L.W.); 2 Animal-Related Injury Treatment Center, Wuhan Jinyintan Hospital, Tongji Medical College and State Key Laboratory for Diagnosis and Treatment of Severe Zoonotic Infectious Diseases, Huazhong University of Science and Technology, Wuhan 430030, China; 3 Department of Trauma Surgery, Emergency Surgery & Surgical Critical Care, Tongji Trauma Center, Tongji Hospital, Tongji Medical College, Huazhong University of Science and Technology, Wuhan 430030, China

**Keywords:** rabies, blood-brain barrier, brain-targeted delivery, antibody engineering, shuttle antibody, immunotherapy

## Abstract

Rabies remains one of the clearest therapeutic paradoxes in infectious diseases: it is largely preventable before neuroinvasion, yet once clinical symptoms appear, mortality approaches 100%. This sharp transition reflects more than delayed diagnosis alone. Wild-type rabies virus reaches and spreads within the central nervous system under conditions of relative immune silence, while the blood–brain barrier (BBB) severely restricts the entry of circulating immune effectors, including virus-neutralizing antibodies. As a result, conventional immunotherapy, although highly effective in post-exposure prophylaxis, performs poorly after symptom onset because it no longer reaches the relevant compartment. This review examines whether engineered antibodies can overcome that limitation and provide a realistic path toward brain-targeted rabies immunotherapy. We first outline why symptomatic rabies remains refractory to standard immune intervention, emphasizing the combined roles of viral immune evasion and BBB-mediated anatomical exclusion. We then review recent proof-of-concept studies showing that antibody-based rescue after central nervous system invasion is biologically plausible, particularly when antibodies are delivered directly into the central nervous system (CNS), retain Fc-dependent immune activity, or are modified to improve BBB penetration. Building on these findings, we discuss key design principles for next-generation therapeutics, including epitope breadth, resistance to viral escape, Fc tuning, and delivery modules based on peptide shuttles or receptor-mediated transcytosis platforms such as TfR1- and CD98hc-targeted systems. Finally, we highlight the major translational barriers that still separate experimental rescue from clinical therapy, including the narrow therapeutic window, model limitations, safety concerns, ethical issues, implementation constraints, and the need for standardized endpoints.

## 1. Introduction

Rabies represents one of the most haunting paradoxes in modern medicine. While nearly 100% preventable if addressed before neuroinvasion, it becomes almost uniformly fatal the moment clinical symptoms emerge [1,2,3]. This is more than just a stark contrast; it is the definitive barrier of the field. We have mastered the art of prevention, yet we remain functionally helpless once the virus breaches the central nervous system (CNS).

Unsurprisingly, the literature reflects the same imbalance. Most rabies reviews still center on vaccination, post-exposure prophylaxis (PEP), rabies immunoglobulin (RIG) replacement, and conventional monoclonal antibodies for passive protection [1,2]. These topics are undeniably important. Even so, they leave a conspicuous gap. Once a patient develops encephalitic rabies, the therapeutic discussion becomes much thinner, often reduced to intensive supportive care, scattered antiviral attempts, and a long record of repeated failure [2,3].

The blood–brain barrier (BBB) lies at the center of this impasse. As a key component of the neurovascular unit, the BBB is designed to restrict paracellular leakage, suppress transcytosis, and limit leukocyte entry into the brain [4]. In many viral encephalitides, BBB dysfunction is itself a major part of the disease process. Rabies is different—at least in its most lethal form. Human imaging studies and animal experiments suggest that during pathogenic rabies infection, the BBB often remains relatively intact until late in the disease course, thereby preventing immune effectors, including virus-neutralizing antibodies (VNAs), from reaching infected CNS tissue in sufficient amounts [3,5]. Wild-type rabies, then, does not merely evade immunity; it exploits an anatomical sanctuary.

This point becomes even clearer when pathogenic and attenuated rabies viruses are compared. Attenuated strains are associated with transient BBB opening, inflammatory cell entry into CNS tissue, and subsequent viral clearance. By contrast, pathogenic strains can induce antiviral immunity in the periphery yet fail to open the BBB, leaving the virus uncleared despite the presence of systemic immune responses [4,5]. Experimental opening of the BBB can partially reverse this logic: once immune effectors are allowed to enter the CNS, otherwise-lethal infection becomes at least biologically more manageable [5]. Similar clues come from rare survivors, in whom antibodies or immune cells were detected in cerebrospinal fluid or other CNS compartments [3,5]. More recently, BBB-penetrating strategies such as SynB1-conjugated antibody cocktails have brought the delivery problem into even sharper focus [6]. In parallel, BBB-shuttle technologies based on platforms such as TfR1- and CD98hc-mediated transport have advanced rapidly in CNS drug-delivery research, providing a practical engineering framework for brain-directed antibody design [7,8,9].

This review is built around that therapeutic shift. Rather than revisiting vaccination or routine PEP, we focus on a narrower question: whether engineered antibodies can be designed to reach the infected brain, retain antiviral activity within the CNS, and thereby provide a plausible path toward brain-targeted rabies immunotherapy. Put simply, the challenge is no longer just to make a stronger anti-rabies antibody. It is to make one that can cross the silent barrier.

## 2. Why Conventional Immunotherapy Fails After Symptom Onset

**Figure 1:**
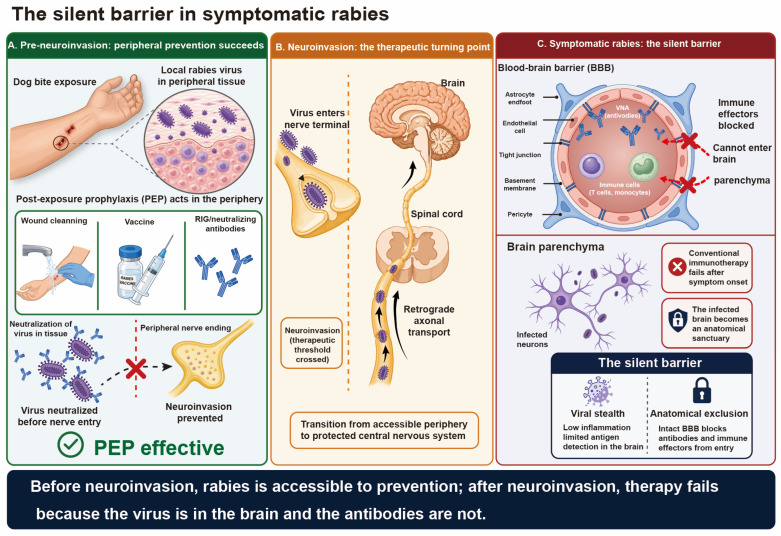
The silent barrier in symptomatic rabies. Before neuroinvasion, the rabies virus remains accessible in peripheral tissues, where wound cleansing, vaccination, and passive immunization can neutralize the virus and prevent entry into peripheral nerves. Once the rabies virus enters neurons and undergoes retrograde axonal transport to the spinal cord and brain, however, the therapeutic problem shifts from the periphery to the central nervous system (CNS). In symptomatic rabies, wild-type virus persists within CNS tissue under conditions of relative immune silence, while the blood–brain barrier (BBB) remains sufficiently restrictive to limit the entry of circulating virus-neutralizing antibodies and other immune effectors. As a result, conventional immunotherapy fails after symptom onset not because antibodies lack antiviral activity, but because they are delivered to the wrong compartment. Black arrows indicate the direction of viral spread and transport; red dashed arrows indicate attempted immune-effector entry; and red crosses indicate blocked passage.

Conventional immunotherapy fails in symptomatic rabies for a simple reason that is easy to state and hard to solve: by the time symptoms appear, the virus is no longer where these therapies work best. PEP, RIG, and standard monoclonal antibodies are designed for the peripheral phase of infection. They are highly effective in this peripheral setting. However, once rabies has crossed into the central nervous system (CNS), the problem changes from peripheral neutralization to intracerebral access. In this sense, the failure of conventional immunotherapy after symptom onset is not simply a failure of antibody potency, but a failure of anatomical access, as shown in Figure 1.

Clinical data already hint at this shift. In the Lancet Infectious Diseases systematic review of 122 fatal breakthrough infections after modern PEP, the median interval from exposure to symptom onset was only 20 days (IQR 16–24), 77% of patients received PEP within 2 days, and 69% had severe wounds, often involving the head, face, or neck. Importantly, although 56% of cases had reported or possible deviations from core PEP practices, 24 patients developed rabies despite completing post-exposure vaccination with no reported deviation, and in 13 of those cases no clear cause could be identified. This is a crucial point: most failures are still explained by exposure severity or imperfect prophylaxis, but a subset cannot be dismissed so easily. In those patients, conventional immunotherapy was not simply absent. It was insufficient [1].

The pathobiology of rabies helps explain why. Wild-type rabies virus does not usually trigger the kind of early, florid neuroinflammation seen in many other viral encephalitides. It moves through neurons, reaches the brain by retrograde axonal transport, and keeps innate alarms relatively subdued [3,10,11]. Experimental work has long shown that attenuated strains and pathogenic strains behave differently here. Wang and colleagues reported that attenuated rabies virus activates host innate immune responses in the CNS, whereas pathogenic virus largely evades them [12]. More recent syntheses of rabies immune escape reach the same conclusion: street strains suppress interferon signaling, delay apoptosis, and blunt the chemokine milieu needed for efficient antiviral clearance [11]. The result is a dangerous mismatch. Peripheral immunity can develop. Brain-directed control still fails.

The blood–brain barrier (BBB) turns that mismatch into a therapeutic bottleneck. Human imaging studies and animal experiments indicate that in lethal rabies the BBB often remains relatively intact until late in disease, limiting the entry of immune effectors into infected CNS tissue [3,5]. Wang and colleagues summarized this sharply: in lethal infection, an intact BBB prevents T cells, B cells, and virus-neutralizing antibodies (VNAs) from reaching the CNS, whereas survivors—often infected with less pathogenic bat-associated variants—show evidence of enhanced BBB permeability and detectable antibodies in cerebrospinal fluid [5]. The old idea that symptomatic rabies is untreatable because it is simply “too late” is therefore incomplete. It is late, yes. But it is also anatomically shielded.

The comparative animal data are even more persuasive. In the Hooper group’s work, 10 rabies virus strains were examined after intradermal infection. Several pathogenic strains, including Thai-DRV, CVS-N2c, and SHBRV, killed mice within about 8–12 days, whereas other pathogenic strains such as DRV-4, CosRV, and skunk RV caused death within 12–17 days. By contrast, the less pathogenic strains ERA, PM, and CVS-F3 also reached the CNS, but infected animals survived. What separated survival from death was not the mere existence of peripheral immunity: all infected animals developed rabies-specific IgG, and neuroinvasive strains induced CNS TNF-α and IL-6 expression. The decisive difference was whether immune effectors gained access to CNS tissue across the BBB [4]. Roy et al. had already shown this in the SHBRV model: failure to open the BBB and deliver immune effectors to the CNS led to lethal outcome, whereas experimentally opening the BBB permitted immune-cell entry, promoted clearance, and prevented death [13,14]. Later work linked this process to chemokine-driven barrier remodeling and tight-junction downregulation, especially involving CXCL10 and related inflammatory programs [15,16]. Together, these data suggest that pathogenic rabies does not merely evade immunity; it also keeps immune effectors outside the infected CNS compartment.

This is where conventional immunotherapy breaks down. RIG and standard monoclonal antibodies can neutralize virus at the wound site or in peripheral tissues, but they penetrate the brain poorly. Experimental studies show that antibody-mediated control improves when BBB permeability is enhanced, when antibodies are delivered directly into the CNS, or when antibody constructs are modified to improve BBB transit [6,17,18]. These findings converge on the same conclusion: after symptom onset, treatment failure reflects both timing and compartmental inaccessibility. Antibodies can still matter, but too little reaches the infected brain, too late.

This is why post-onset therapy needs a different therapeutic logic from PEP. In the peripheral phase, the goal is to neutralize the virus before neuroinvasion. In symptomatic rabies, the therapeutic problem is different: the antibody must reach infected CNS tissue, retain antiviral activity in that compartment, and, in some settings, cooperate with host immune mechanisms [19]. The failure of conventional immunotherapy is therefore not a reason to abandon antibodies, but a reason to redesign them for the anatomical and immunological constraints of established CNS infection.

## 3. Why Antibodies Still Matter in Symptomatic Rabies

### 3.1. Biological Rationale for Antibody-Based Rescue

It is easy to assume that once rabies reaches the brain, antibodies have already missed their window. That conclusion is understandable, but it is too blunt [20]. Rabies virus glycoprotein (RABV-G) remains the most compelling therapeutic target even in late disease: it is the only virion-surface protein, it is essential for receptor engagement and membrane fusion, and it is the dominant target of virus-neutralizing antibodies (VNAs) [2,10,11]. So, the problem is not that antibodies have become biologically irrelevant. The problem is that, in symptomatic rabies, the right antibodies rarely reach the right compartment in sufficient quantity or with the right effector profile.

That distinction matters. Antibodies are not just neutralizers; they are engineerable biologics. Epitope breadth, Fc function, half-life, and delivery strategy can all be tuned. In other words, the field is no longer limited to asking whether antibodies work. The more useful question is what kind of antibody can still work after CNS invasion [2,13].

### 3.2. What Proof-of-Concept Studies Have Shown

The strongest argument for keeping antibodies in play comes from the rescue studies themselves. de Melo and colleagues built a stringent field-virus mouse model in which virus was detectable in the spinal cord by 4 days post-infection (dpi), in the brain by 5 dpi, motor impairment began at 7 dpi, typical clinical signs appeared from 8 dpi, and all untreated animals died between 10 and 13 dpi [18]. In that setting, a single intramuscular RVC20/RVC58 cocktail was not enough: even at 20 + 20 mg/kg, treatment at 6 dpi rescued only 1 of 5 animals, and later peripheral therapy failed. The picture changed once the same cocktail was given both intramuscularly and by continuous intracerebroventricular infusion. Survival was 100% when treatment started at 6 dpi, 55.6% (5/9) at 7 dpi, and 33.3% (5/15) at 8 dpi. That was not a modest signal. It was proof of principle.

The next question was whether antibodies could still work without direct CNS infusion. Mastraccio et al. moved the field forward by testing a single peripheral dose of the human mAb F11 against established CNS-resident lyssavirus infection. In ABLV-luc infection, one 10 mg/kg dose given at day 5 or day 7 post-infection produced 100% and 83% survival, respectively, even though the virus was already replicating robustly in the CNS by then. In the more stringent CVS-11 model, F11 still produced resolution of disease signs and long-term survival in 67% of day-5-treated mice and 50% of day-7-treated mice, whereas all untreated mice died by days 8–11 [19]. Just as important, the mechanism was not simple neutralization. F11 lost durable efficacy in mice lacking adaptive immunity; B cells were dispensable, but CD4 T cells were required, CD8 T cells were not essential for survival, and disruption of Fcγ receptor binding reduced protection. So the antibody was doing more than binding virus. It was reshaping the host response inside the infected CNS.

Then came the BBB-delivery study. Ren et al. showed that a SynB1-conjugated triple-antibody cocktail targeting three distinct RABV-G epitopes produced 80% survival when treatment began at 5 dpi, 40% at 6 dpi, and 0% at 7 dpi. Under the same conditions, the unconjugated human–mouse chimeric cocktail achieved only 20% survival against CVS and 0% against dog-originated rabies virus, while a single SynB1-conjugated antibody was clearly weaker than the triple cocktail. In surviving mice, viral genomic RNA in the spinal cord, brainstem, cerebellum, and cerebrum fell markedly, RABV-positive cells on immunohistochemistry became scarce, and neuroinflammatory markers such as TNF-α, IFN-γ, CXCL10, and CCL5 were attenuated [6]. Again, the message was concrete rather than theoretical: brain delivery changes outcome.

### 3.3. Core Interpretation of the Current Evidence

Put together, these studies say something quite specific. Symptomatic rabies is not absolutely antibody-refractory. It is delivery-limited and mechanism-limited. Antibodies still matter because they target the right viral structure, but successful rescue now appears to require at least one of three things: direct CNS access, improved BBB transit, or Fc- and T-cell-dependent cooperation that turns neutralization into true intraparenchymal control [15,18,19].

## 4. Engineering Antibodies for Brain-Targeted Rabies Immunotherapy

### 4.1. Designing the Antiviral Antibody

**Figure 2:**
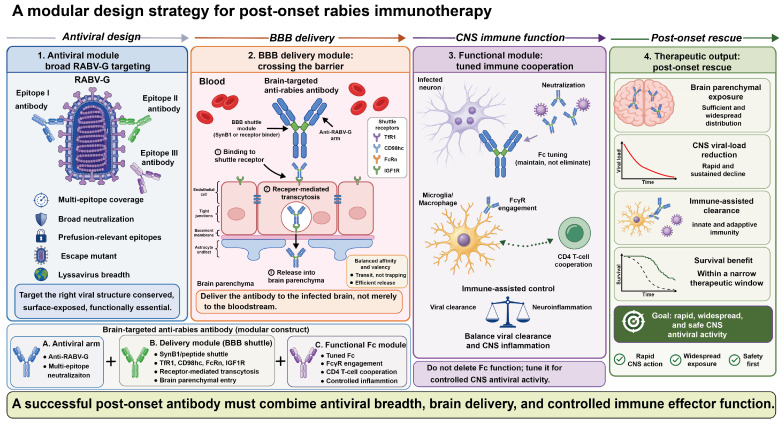
Engineering blueprint for brain-targeted anti-rabies antibodies. A next-generation antibody for post-onset rabies immunotherapy should be designed as a modular construct rather than as a conventional peripheral neutralizing antibody. The antiviral module should target conserved and functionally essential regions of rabies virus glycoprotein (RABV-G), ideally through multi-epitope recognition to broaden coverage and reduce viral escape. The delivery module should enable antibody transport across the blood-brain barrier (BBB), potentially through peptide shuttles or receptor-mediated transcytosis platforms such as TfR1-, CD98hc-, FcRn-, or IGF1R-based systems. Once in the infected central nervous system, the functional module should retain appropriately tuned Fc-mediated activity to support immune-assisted viral control while avoiding excessive neuroinflammation. The desired therapeutic output is rapid and sufficiently widespread brain parenchymal exposure, reduction in CNS viral burden, immune-assisted clearance, and survival benefit within the narrow therapeutic window of symptomatic rabies. Arrows indicate the direction of antibody transport, viral neutralization, immune-cell cooperation, and progression through the modular design.

If this field is going to move beyond proof of concept, the antibody itself has to be treated as a deliberately engineered therapeutic object, not as a passive extension of PEP logic. The starting point is still rabies virus glycoprotein (RABV-G). That has not changed. It is the only virion-surface protein, it governs receptor engagement and membrane fusion, and it remains the dominant target of protective neutralizing antibodies [2,10,20]. What has changed is the design criterion. In symptomatic rabies, potency alone is not enough. Figure 2 summarizes the proposed engineering blueprint for brain-targeted anti-rabies antibodies, integrating antiviral specificity, BBB delivery, Fc-mediated immune cooperation, and therapeutic outcomes.

Breadth is the first non-negotiable feature. One reason is obvious: a single-epitope antibody is easier for the virus to escape. The other is more practical. A clinically useful rescue therapeutic has to cover laboratory strains, dog-originated street strains, and ideally a broader slice of phylogroup I lyssaviruses. The de Melo study made this logic concrete. RVC20 and RVC58 were not chosen simply because they neutralized rabies; they recognized two distinct antigenic sites on RABV-G and, when used together, produced rescue where single-compartment therapy failed [18]. Later mechanistic work sharpened the picture: RVC58 was the stronger extracellular neutralizer, whereas RVC20 was more effective at limiting spread from infected cells and showed stronger FcγR engagement [21].

Structural data help explain why some antibodies are better building blocks than others. Hellert and colleagues showed that RVC20 binds a highly conserved surface on domain III of RABV-G and locks the glycoprotein in its prefusion conformation, thereby blocking the acid-triggered rearrangement needed for fusion [22]. Callaway et al. extended this broader concept by resolving prefusion trimeric RABV-G bound to a potently neutralizing human antibody, reinforcing the idea that prefusion-specific recognition is not merely structurally elegant but therapeutically relevant [23]. For antibody engineering, that matters. An antibody that recognizes a conserved prefusion epitope is doing more than occupying space; it is interrupting a required conformational step in viral entry.

The 2025 SynB1 study offers a second design lesson: epitope breadth and brain delivery should be optimized together. Although the lead single antibody showed strong binding and neutralizing activity, the multi-epitope cocktail performed better in delayed-treatment models after BBB-shuttle conjugation. This suggests that a post-onset rabies antibody should not be designed around potency alone. Breadth, escape resistance, and delivery compatibility are likely to matter together [6].

### 4.2. Engineering Brain Delivery

This is where the field becomes genuinely different from classical rabies immunotherapy. The question is no longer just which antibody to use. It is how to get it into brain parenchyma without causing more trouble than it solves.

Direct CNS delivery provides the clearest demonstration that anatomical access can change the outcome of antibody therapy after CNS invasion [18]. However, its main value is conceptual rather than practical. It shows that delivery, not only neutralization, is a limiting variable in post-onset rabies. Once CNS access is achieved, the remaining challenge is not simply to increase antibody dose, but to match exposure, timing, distribution, and immune function within the infected brain.

The problem, of course, is that ICV delivery is not the scalable answer for human rabies. It proves the principle, but it does not solve the platform problem. That is why BBB-shuttle design matters.

The most mature route is receptor-mediated transcytosis (RMT), especially through TfR1 and CD98hc [8,9,24,25]. The classic TfR lesson is that valency and affinity must be balanced rather than maximized. In the Hultqvist study, a TfR-binding shuttle was engineered so that the antibody interacted monovalently with the receptor despite having two TfR-binding modules. That design produced brain concentrations of 2–3% of the injected dose per gram of brain just 2 h after administration—about 80-fold higher than unmodified mAb158—and by 3 days the fusion protein concentration in AD-transgenic brains was 9-fold higher than in wild-type brains [26]. The message is straightforward: efficient BBB transit does not come from grabbing the receptor as tightly as possible; it comes from binding in a way that still allows release.

CD98hc offers a different profile. Chew et al. showed that CD98hc-targeted antibody transport vehicles achieved significantly elevated parenchymal exposure at all measured time points, reaching roughly 20–25-fold over control human IgG at 14 days after a single dose [8]. High-magnification histology showed a transition from vascular staining at day 1 to diffuse parenchymal staining by day 7, and repeated weekly dosing at 50 mg/kg led to additional brain accumulation without measurable effects on circulating monocytes, lymphocytes, or reticulocytes [8]. Across a 20–550 nM affinity range, brain exposure changed little, although very weak binding began to reduce uptake in some bivalent variants [8]. These findings are relevant to rabies antibody design because CD98hc may offer a transport profile distinct from TfR1 and receptor choice could influence both brain exposure and peripheral distribution.

Rabies-specific work is now beginning to translate these general BBB principles into disease-focused constructs. In the SynB1 paper, four BBB-penetrating peptides were compared head-to-head after conjugation to the lead antibody 7A3-H. SynB1 outperformed RVG, TGN, and THR in brain fluorescence readouts, and confocal imaging 24 h after dosing showed SynB1-7A3-H colocalizing with neurons and RABV-G but not obviously with blood vessels, arguing that the construct had crossed the BBB rather than merely sticking to it [6]. In the same study, the unconjugated three-antibody cocktail had a half-life of 7.85 days, whereas SynB1-conjugated antibodies showed kidney and liver half-lives of 4.48 and 4.60 days, respectively, with no significant biochemical abnormalities or histologic lesions detected 28 days later [6]. These findings do not constitute a complete safety package, but they support peptide-assisted delivery as a credible engineering strategy for rabies antibody development.

### 4.3. Tuning Antibody Effector Function

A brain-deliverable antibody that only neutralizes extracellular virions may still underperform once CNS infection is established. The rescue studies keep returning to the same point: Fc biology matters.

Fc-engineering experiments support this point. In the de Melo study, Fc-silenced antibodies retained neutralizing activity but lost much of their rescue capacity when treatment was delayed, indicating that viral binding alone was not enough once CNS infection was established [18]. Mastraccio et al. reached a similar conclusion from a different model. F11-mediated control of CNS-resident lyssavirus infection depended on host adaptive immunity, particularly CD4 T cells, and was weakened when FcγR binding was disrupted despite preserved in vitro neutralization [19]. These findings suggest that the most useful antibody for symptomatic rabies may be one that combines neutralization with carefully retained immune cooperation.

That creates a real engineering tension. Strong Fc effector function may help viral clearance, microglial engagement, or T-cell-supported control, but it may also increase the risk of neuroinflammation. The de Melo and F11 studies suggest that complete Fc silencing is unlikely to be optimal. They do not imply that every effector-positive IgG will be safer or more effective. Rather, they support Fc tuning, rather than Fc deletion, as a more rational design strategy.

### 4.4. Design Principles for Post-Onset Therapy

The available evidence does not yet define a single optimal antibody product for symptomatic rabies. It does, however, point to several design principles that should guide the next stage of development.

The first requirement is anatomical access. Conventional antibody therapy fails after symptom onset largely because circulating antibodies do not reach the infected CNS in sufficient amounts. Studies using direct CNS delivery, BBB permeability modulation, and BBB-shuttle conjugation all support the same conclusion: after neuroinvasion, antibody potency has little value unless the antibody reaches the compartment where viral replication is occurring [6,13,14,15,16,17,18]. This does not mean that brain entry alone is enough. CNS exposure must be timely, sufficiently distributed across relevant brain regions, and paired with an antibody that can act against the viral target under the conditions of established infection.

The antiviral component should therefore be designed for breadth as well as potency. RABV-G remains the central target because it is exposed on the virion surface and is required for receptor engagement and membrane fusion [2,10,20]. For post-onset therapy, however, a strong neutralization titer in vitro is not sufficient as a design endpoint. Antibodies should preferably recognize conserved and functionally constrained regions of RABV-G, especially epitopes linked to the prefusion state or fusion machinery, because such sites are less permissive to viral escape [21,22,23]. Multi-epitope coverage is also important. In a rescue setting, viral replication has already reached the nervous system, and a single-antibody strategy may be more vulnerable to incomplete coverage or escape. A cocktail or multispecific format may therefore be more appropriate than a single highly potent neutralizer, provided that CNS delivery can be achieved.

A third principle is that Fc function should be tuned rather than removed by default. The purpose of a brain-targeted antibody is not only to neutralize free virions. Established CNS infection may also require immune-assisted clearance of infected cells or infected tissue compartments. The de Melo and Mastraccio studies both suggest that Fc-dependent mechanisms can contribute to protection, even when neutralizing activity is preserved [18,19]. At the same time, the inflamed CNS is not a neutral environment. Excessive Fc-mediated activation could worsen neuroinflammation, whereas complete Fc silencing could weaken viral control. Future constructs should therefore treat Fc biology as an adjustable therapeutic variable, balancing FcγR engagement, complement risk, microglial activation, and CD4 T-cell cooperation.

The delivery module should be selected with the therapeutic window in mind. Rabies progresses rapidly once clinical disease begins, so a platform that crosses the BBB slowly, weakly, or inconsistently may not be useful even if it is elegant from an engineering perspective. Direct CNS delivery proves the importance of access but is invasive and difficult to scale [18]. Peptide shuttles such as SynB1 offer rabies-specific proof of concept, but dosing, pharmacokinetics, tissue distribution, and safety still require further validation [6,27]. Receptor-mediated transcytosis systems such as TfR1- and CD98hc-based platforms provide a more generalizable delivery logic, but their performance will depend on receptor affinity, valency, endothelial trafficking, peripheral binding, and disease-state effects on the BBB [7,8,9,24,25,26]. These parameters should not be optimized in isolation. A delivery system that maximizes receptor binding but fails to release into brain parenchyma, or one that increases brain exposure at the cost of unacceptable systemic binding, would not solve the clinical problem.

These considerations suggest a practical benchmark for future studies. Candidate therapies should be evaluated not only by serum neutralization titers or survival curves, but also by brain exposure, regional CNS viral burden, time to treatment, neurologic function, inflammatory injury, and post-survival sequelae. The most informative models will be those in which treatment begins after confirmed CNS invasion and is tested against clinically relevant street-virus challenge, rather than only under early or permissive conditions.

The evidence now points toward a more integrated design strategy. A plausible post-onset antibody therapy should combine escape-resistant RABV-G recognition, adequate and controlled CNS delivery, preserved but restrained immune-effector function, and a safety profile suitable for acute encephalitic disease. The goal is not simply to build a stronger neutralizing antibody or to open the BBB more aggressively. It is to match antiviral activity, brain access, timing, and immune cooperation within the narrow biological window in which symptomatic rabies may still be modifiable.

## 5. Translational Strategies and Development Priorities

### 5.1. Most Plausible Therapeutic Architectures

Future brain-directed rabies immunotherapy is likely to draw on both rabies-specific rescue studies and the broader BBB drug-delivery field. The main candidate architectures include direct CNS delivery, systemic Fc-functional monoclonal antibodies, peptide-shuttle antibodies, receptor-mediated transcytosis platforms, and controlled BBB opening. Table 1 summarizes their main rationale, limitations, risks, and supporting evidence.

Among these approaches, direct CNS delivery provides the clearest proof that anatomical access can determine whether antibody therapy remains effective after CNS invasion.

However, because of its invasive nature, this approach should be reserved for carefully selected patients in exceptional rescue situations [18]. Systemic Fc-functional monoclonal antibodies offer a less invasive architecture and highlight the importance of immune cooperation. The key lesson from this approach is that neutralization alone may not be sufficient; FcγR engagement and CD4 T-cell-dependent mechanisms may also contribute to control of CNS-resident infection [19]. Peptide-shuttle antibodies, represented by SynB1-conjugated constructs, provide rabies-specific proof of concept for systemic enhancement of brain entry [6]. This approach is attractive because it links antiviral breadth with delivery engineering, but its dose, pharmacokinetics, durability, and safety remain early-stage issues [6,27]. TfR1- and CD98hc-based receptor-mediated transcytosis platforms extend the delivery concept beyond rabies-specific peptide shuttles. These systems may support modular antibody engineering, but they still need validation in rabies rescue models and careful assessment of receptor binding, peripheral distribution, and disease-state variability [7,8,9,24,25,26]. Controlled BBB opening remains important mainly as a mechanistic lesson. It shows that CNS access can affect viral clearance, but its non-specific nature and inflammatory risks make it less attractive as a stand-alone clinical strategy [13,14,15,16,17].

### 5.2. Major Translational Barriers

The first barrier is the therapeutic window. Across both direct CNS delivery and BBB-shuttle studies, efficacy declines sharply when treatment is delayed after CNS invasion [6,18]. This creates a practical problem for human rabies, in which exposure recognition, diagnosis, referral, and confirmation of CNS involvement may all occur late. A clinically useful rescue therapy would therefore need to act rapidly, achieve measurable CNS exposure, and remain effective in the setting of established neurologic disease.

The second barrier is model generalizability. Existing rescue studies have used different viruses, routes of infection, treatment windows, and outcome measures, including Tha-RABV, CVS-11, ABLV-luc, and dog-originated rabies virus models [6,18,19]. These systems are valuable, but rescue in one model does not guarantee efficacy across street-virus infection, different exposure sites, or more advanced neurologic disease. Future development should therefore prioritize delayed-treatment models using clinically relevant dog-originated strains, objective confirmation of CNS invasion before treatment, and standardized readouts across spinal cord, brainstem, cerebellum, and cerebrum.

The third barrier is delivery-related safety. Direct CNS delivery proves that anatomical access can change therapeutic outcome, but it is invasive and requires specialized neurosurgical and intensive-care support [18]. BBB-shuttle approaches are more attractive for systemic administration, but they introduce different uncertainties, including altered pharmacokinetics, peripheral tissue distribution, immunogenicity, receptor saturation, and off-target exposure [6,7,8,9,24,25,26,27]. Early animal safety signals are encouraging, but they are not sufficient to de-risk repeated dosing, inflamed CNS tissue, or critically ill patients with encephalitic disease.

A final barrier is the public-health boundary of any rescue biologic. Even if a brain-targeted antibody becomes clinically testable, it would remain a specialized intervention for selected patients rather than a substitute for prevention. Population-level rabies control still depends on wound care, timely PEP, rabies immunoglobulin when indicated, dog vaccination, surveillance, and integrated bite-case management [28]. For this reason, post-onset antibody engineering should be framed as a rescue complement to established prevention strategies, not as an alternative to them.

### 5.3. What the Field Should Do Next

Three priorities follow from the current evidence. The first is to build better rescue models. Future studies should use clinically relevant dog-originated strains, delayed treatment after objective confirmation of CNS invasion, and longitudinal endpoints beyond survival. These endpoints should include viral RNA in the spinal cord and specific brain regions, immunohistochemistry, neurologic function, body weight, inflammatory injury, and long-term sequelae [6,18,19].

The second priority is a true head-to-head comparison of delivery platforms. Ideally, the same anti-rabies antibody or antibody cocktail should be tested across no-shuttle, peptide-shuttle, TfR1-shuttle, and CD98hc-shuttle formats in the same infection model. At present, payload and delivery strategy are often changed at the same time, making it difficult to determine whether improved efficacy reflects antibody specificity, epitope breadth, brain delivery, Fc function, or a combination of these factors [6,7,8,9,24,25,26,27].

**Figure 3:**
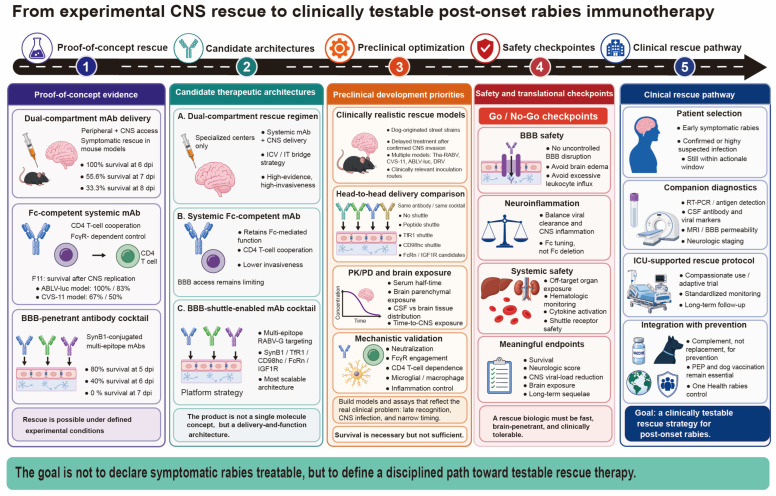
Translational roadmap from proof-of-concept evidence to clinical rescue. Brain-targeted antibody therapy for symptomatic rabies must progress through a staged translational pathway. Current proof-of-concept studies indicate that rescue is biologically possible when antibodies gain access to the infected central nervous system, retain appropriate Fc-mediated activity, or are engineered to improve blood–brain barrier penetration. These findings support three candidate therapeutic architectures: dual-compartment rescue regimens, systemically administered Fc-competent monoclonal antibodies, and BBB-shuttle-enabled multi-epitope antibody constructs. Future development should prioritize clinically realistic delayed-treatment models, head-to-head comparison of delivery platforms, pharmacokinetic and pharmacodynamic assessment of brain exposure, and mechanistic validation of neutralization, FcγR engagement, CD4 T-cell cooperation, and neuroinflammatory control. Before clinical deployment, candidate therapies must pass safety checkpoints related to BBB integrity, neuroinflammation, systemic toxicity, and meaningful functional endpoints. Ultimately, post-onset rescue biologics should be developed as ICU-supported, carefully monitored interventions for selected patients, while remaining complementary to post-exposure prophylaxis, dog vaccination, and broader One Health rabies prevention. Arrows indicate progression through the staged translational pathway from proof-of-concept rescue to clinical implementation.

The third priority is to define translational endpoints that are relevant to human rescue therapy. These should include measurable CNS exposure, reduction in viral burden in anatomically relevant CNS regions, neurologic improvement, acceptable systemic safety, and evidence that immune-effector functions are helpful rather than harmful. If Fc function and CD4 T-cell-dependent cooperation are part of efficacy, they should be optimized rather than engineered away [18,19]. Figure 3 outlines this translational roadmap, from proof-of-concept evidence to a clinically testable rescue pathway.

### 5.4. Safety, Ethics, and Implementation

Any post-onset antibody strategy for rabies will have to be judged not only by antiviral activity, but also by whether it can be delivered safely in patients with acute encephalitic disease. The main approaches discussed above carry different risks. Direct CNS delivery gives the clearest route to brain exposure, but it requires neurosurgical access and intensive monitoring. Infection, bleeding, catheter-related complications, intracranial pressure changes, and local tissue injury would all be relevant concerns [18]. Controlled BBB opening raises a different problem. It may increase the entry of antibodies and immune effectors, but it is not selective for antiviral molecules. Edema, uncontrolled leukocyte trafficking, and worsening neuroinflammation would need to be considered [13,14,15,16,17]. Peptide-shuttle and receptor-mediated transcytosis platforms are less invasive, but they introduce other uncertainties, including altered pharmacokinetics, peripheral tissue binding, immunogenicity, receptor competition, and species-dependent transport differences [6,7,8,9,24,25,26,27].

The ethical issues are also unusually difficult. Symptomatic rabies is almost uniformly fatal, but many patients present with agitation, encephalitis, impaired consciousness, or rapidly progressive neurologic failure. Direct informed consent may therefore be impossible in the very population for whom rescue therapy would be considered. Future first-in-human CNS rescue studies would require legally authorized representative consent, independent ethical review, and a clear risk–benefit framework [29,30]. Because the likelihood of benefit may fall rapidly as neurologic injury progresses, these studies should also define futility thresholds in advance. Such thresholds could include duration of symptoms, coma or brainstem involvement, evidence of irreversible neurologic injury, uncontrolled organ failure, and inability to achieve timely CNS drug exposure.

Practical feasibility may be an even larger barrier than product design. Even for established rabies prevention, access to vaccination, rabies immunoglobulin, procurement systems, and timely post-exposure prophylaxis can be constrained in high-burden settings [31,32]. These barriers would likely be greater for multi-epitope antibodies and BBB-shuttle constructs, which may be expensive to manufacture, store, and distribute. Direct CNS delivery would add further requirements, including specialist centers, trained personnel, imaging support, and close neurologic monitoring. For these reasons, post-onset rescue biologics should be viewed as highly specialized interventions for selected patients, not as substitutes for wound care, vaccination, rabies immunoglobulin, dog vaccination, or broader One Health prevention [28,31].

Combination therapy may eventually be necessary, but it should be designed carefully. A CNS-penetrant antibody could reduce viral burden, whereas antiviral small molecules, BBB-protective strategies, or selected neuroinflammatory modulators might address other parts of established encephalitic disease [2,3]. However, broad immunosuppression would be risky. FcγR engagement and CD4 T-cell-dependent cooperation may contribute to viral control, so any anti-inflammatory approach would need to reduce damaging neuroinflammation without blocking useful antiviral immunity [18,19]. At present, combination therapy should be treated as a development priority rather than a clinically defined regimen.

Patient stratification is another unresolved issue. BBB-shuttle systems should not be assumed to behave identically across all patients. TfR1, CD98hc, and other transport receptors may vary with age, endothelial activation, inflammation, genetic background, species, and disease stage [7,8,9,24,25,26]. This variability could affect both CNS exposure and peripheral toxicity. Future studies may therefore need companion diagnostics or stratification tools, such as neuroimaging of BBB integrity, CSF viral and immune markers, systemic inflammatory profiling, and pharmacokinetic evidence of CNS exposure. These requirements do not weaken the rationale for brain-targeted antibodies. They define the conditions under which such therapies can be tested responsibly.

## 6. Conclusions: Breaking the Silent Barrier

Symptomatic rabies remains one of the most difficult therapeutic settings in infectious disease. Available evidence suggests that treatment failure is not explained by timing alone. It also reflects a compartment problem: pathogenic rabies virus can persist in the CNS while circulating antibodies and immune effectors remain largely excluded by the BBB.

Recent preclinical studies have challenged the view that post-onset rabies is completely antibody-refractory. Experimental rescue has been observed when antibodies gain direct CNS access, retain useful immune-effector function, or are engineered to improve BBB transit. These findings remain proof-of-concept evidence, but they clarify the design problem. Future therapies will need to combine escape-resistant RABV-G recognition, controlled CNS delivery, appropriately tuned Fc-mediated cooperation, and safety standards suitable for acute encephalitic disease.

The central challenge is therefore no longer simply to make a more potent neutralizing antibody. It is to design an antibody-based intervention that can reach the infected brain, act within a narrow therapeutic window, and do so without worsening CNS injury. Brain-targeted antibody therapy should be developed as a carefully monitored rescue strategy for selected patients, while prevention through wound care, vaccination, rabies immunoglobulin, dog vaccination, and One Health programs remains the foundation of rabies control.

## Figures and Tables

**Table 1:** Comparative overview of brain-targeted antibody strategies for symptomatic rabies.

Strategy	Key Rationale	Limitations and Risks	References
Direct CNS delivery/ICV infusion	Bypasses the BBB; confirms CNS access as a key bottleneck.	Invasive; requires neurosurgical and ICU support. Risks: infection, bleeding, pressure changes, local injury.	[18]
Systemic Fc-functional mAbs	Systemic dosing with retained FcγR and CD4 T-cell cooperation.	Depends on BBB status, timing, viral burden, and host immunity. Fc activity may aid clearance but also increase inflammation.	[19]
SynB1/peptide-shuttle antibodies	Peptide-assisted BBB entry; rabies-specific proof of concept.	Early-stage. Dose, PK, durability, cost, and cold chain needs remain unresolved. Risks: off-target exposure, immunogenicity.	[6,27]
TfR1/CD98hc RMT shuttles	Uses BBB transport receptors; modular platform for antibody delivery.	Not tested in rabies. Receptor expression may vary; risks include peripheral binding and altered biodistribution.	[7,8,9,24,25,26]
Controlled BBB opening	Permits circulating antibodies and immune effectors to enter the CNS.	Non-specific and hard to control. Risks: edema, leukocyte influx, neuroinflammation, barrier injury.	[4,5,13,14,15,16,17]

Abbreviations: BBB, blood–brain barrier; CNS, central nervous system; CD98hc, CD98 heavy chain; FcγR, Fc gamma receptor; ICV, intracerebroventricular; mAbs, monoclonal antibodies; PK, pharmacokinetics; RMT, receptor-mediated transcytosis; TfR1, transferrin receptor 1.

## Data Availability

Data availability is not applicable to this article as no new data were created or analyzed in this study.

## Abbreviations

The following abbreviations are used in this manuscript:

ABLV-luc	Luciferase-expressing Australian bat lyssavirus
AD	Alzheimer’s disease
BBB	Blood–brain barrier
CCL5	C-C motif chemokine ligand 5
CD4	Cluster of differentiation 4
CD8	Cluster of differentiation 8
CD98hc	CD98 heavy chain
CNS	Central nervous system
CosRV	Coyote street rabies virus
CVS	Challenge virus standard
CVS-11	Challenge virus standard 11
CVS-F3	Challenge virus standard F3
CVS-N2c	Challenge virus standard N2c
dpi	Days post-infection
DRV	Dog-related rabies virus
ERA	Evelyn–Rokitnicki–Abelseth strain
F11	F11 monoclonal antibody
Fc	Fragment crystallizable region
FcγR	Fc gamma receptor
IBCM	Integrated bite-case management
ICU	Intensive care unit
ICV	Intracerebroventricular
IFN-γ	Interferon gamma
IgG	Immunoglobulin G
IL-6	Interleukin-6
IQR	Interquartile range
mAb	Monoclonal antibody
PEP	Post-exposure prophylaxis
PM	PM strain
RABV	Rabies virus
RABV-G	Rabies virus glycoprotein
RIG	Rabies immunoglobulin
RMT	Receptor-mediated transcytosis
RNA	Ribonucleic acid
RV	Rabies virus
RVG	Rabies virus glycoprotein-derived peptide
RVC20	RVC20 monoclonal antibody
RVC58	RVC58 monoclonal antibody
SHBRV	Silver-haired bat rabies virus
SynB1	SynB1 peptide
TfR	Transferrin receptor
TfR1	Transferrin receptor 1
TGN	TGN peptide
Thai-DRV	Thailand dog-related rabies virus
THR	THR peptide
TNF-α	Tumor necrosis factor alpha
VNAs	Virus-neutralizing antibodies
